# *PAX5A* and *PAX5B* isoforms are both efficient to drive B cell differentiation

**DOI:** 10.18632/oncotarget.26003

**Published:** 2018-08-28

**Authors:** Charlotte Cresson, Sophie Péron, Laura Jamrog, Nelly Rouquié, Nais Prade, Marine Dubois, Sylvie Hébrard, Stéphanie Lagarde, Bastien Gerby, Stéphane J.C. Mancini, Michel Cogné, Eric Delabesse, Laurent Delpy, Cyril Broccardo

**Affiliations:** ^1^ Inserm, UMR1037 CRCT, F-31000, Université Toulouse III-Paul Sabatier, UMR1037 CRCT, Oncopole, F-31000 Toulouse, France; ^2^ Université de Limoges-CNRS UMR 7276, F-87025 Limoges, France; ^3^ Inserm, UMR1037 CRCT, F-31000, Université Toulouse III-Paul Sabatier, UMR1037 CRCT, Toulouse Hospital University, Oncopole, CS 53717, F-31000 Toulouse, France; ^4^ Aix Marseille Univ, CNRS, INSERM, Institut Paoli-Calmettes, CRCM, F-13009 Marseille, France; ^5^ Université de Limoges-CNRS UMR 7276, Institut Universitaire de France, F-87025 Limoges, France

**Keywords:** Pax5, transcription factor, B cell development, B cells, acute lymphoblastic leukemia

## Abstract

Pax5 is the guardian of the B cell identity since it primes or enhances the expression of B cell specific genes and concomitantly represses the expression of B cell inappropriate genes. The tight regulation of *Pax5* is therefore required for an efficient B cell differentiation. A defect in its dosage can translate into immunodeficiency or malignant disorders such as leukemia or lymphoma.

*Pax5* is expressed from two different promoters encoding two isoforms that only differ in the sequence of their first alternative exon. Very little is known regarding the role of the two isoforms during B cell differentiation and the regulation of their expression. Our work aims to characterize the mechanisms of regulation of the expression balance of these two isoforms and their implication in the B cell differentiation process using murine *ex vivo* analyses. We show that these two isoforms are differentially regulated but have equivalent function during early B cell differentiation and may have functional differences after B cell activation. The tight control of their expression may thus reflect a way to finely tune Pax5 dosage during B cell differentiation process.

## INTRODUCTION

The commitment of hematopoietic stem cells to each cell lineage is strictly controlled. Specific extracellular stimuli and transcription factors play important roles in the development of B cells from hematopoietic stem cells. The transcription factors SPI1 (PU.1) and IKZF1 (Ikaros) work together during the early developmental stages, while TCF3 (E2A), Early B cell Factor 1 (EBF1) and Paired box 5 (PAX5) are crucial from pro-B cells to mature B cell stages [1]. PAX5 is one of the 9 members (PAX1 to PAX9) of the highly conserved paired-box (PAX) domain family of transcription factors [2, 3] characterized by a conserved PAIRED DNA-binding domain at the N-terminal part of the protein and involved in the regulation of tissue homeostasis.

During hematopoiesis, *PAX5* is expressed from the pro-B cell stage and has to be turned off to allow plasma-cell transition [4]. PAX5 is crucial for the maintenance of the B lymphoid lineage identity [5, 6] and for suppression of alternative lineage choices [1, 7]. PAX5 also enhances the transcription of B cell specific genes and participates in the chromatin-remodeling of the immunoglobulin heavy chain (IGH) locus, ensuring its contraction during VDJ recombination [8]. At later stages, PAX5 regulates the IGH 3′ regulatory region (3′RR). The 3′RR is a 30 kb-long cis-acting regulation element of the immunoglobulin heavy chain (IGH) locus containing four enhancers in mice (hs1,2, hs3a, hs3b and hs4) with a strict B lineage specificity. They have been implicated in the late stages of B cell differentiation with a crucial role in class switch recombination (CSR) and somatic hypermutation (SHM) [9–12].

*Pax5* homozygous inactivation in mouse leads to a blockade at the pro-B cell stage [6]. *Pax5*-deficient pro-B cells are able to transdifferentiate into other cell types, such as T, natural killer, and dendritic cells [1, 13] while mature B cells can dedifferentiate into lymphoid precursor cells upon *Pax5* loss even at late stages of B cell differentiation as shown by *Pax5* conditional inactivation [14].

In vertebrates, *PAX5* expression is controlled by two distinct promoters: a distal P1a and a proximal P1b [15] which initiate transcription from two alternative 5′ first exons (exons 1A and 1B respectively) leading to the expression of two isoforms, *PAX5A* and *PAX5B*. The only structural difference between PAX5A and PAX5B consists in a short different N-terminal sequence. Exon 1A encodes 15 amino acids that differ totally from the 14 amino acids encoded by the exon 1B sequence [16]. Despite high sequence similarities between these two isoforms, they show different patterns of expression: *PAX5B* is transcribed in B cells, central nervous system and testis, while *PAX5A*, whose product is also named B cell–specific activator protein (BSAP) is restricted to the B cell lymphoid lineage [4]. However their respective roles in B cell differentiation have never been determined. Our work aims to detail the mechanisms that control the expression of *PAX5A* and *PAX5B* isoforms along B cell development and their effect on B cell differentiation.

## RESULTS

### *Pax5* expression in B cell differentiation is independent of adjacent genes

**Figure 1:**
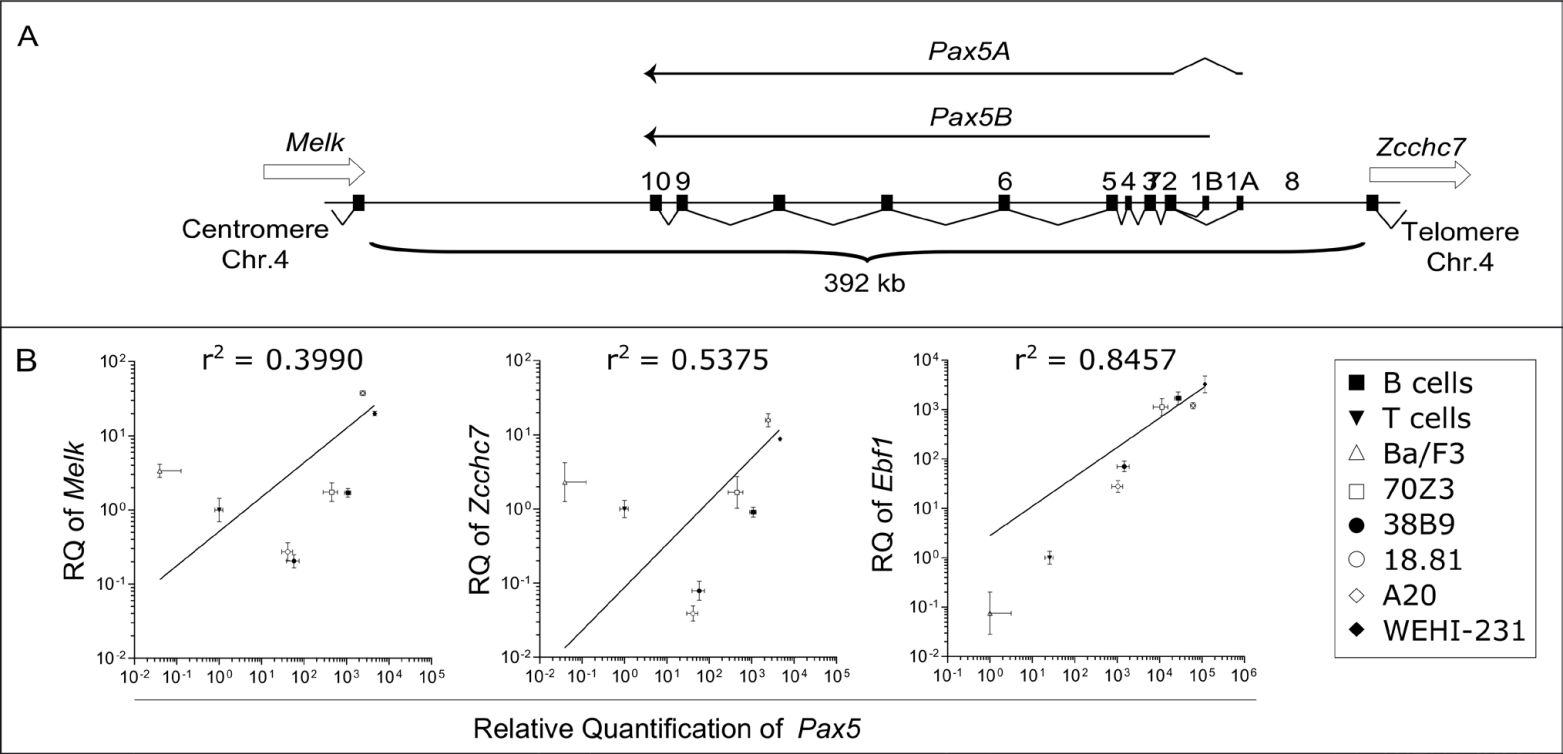
Expression of *Pax5* isoforms is independent of the expression of neighboring genes (**A**) Schematic organization of the genomic region of murine *Pax5* gene. *Pax5* is composed of 11 exons, the first two (exons 1A and 1B) being alternatively used to generate two isoforms (*Pax5A* and *Pax5B* respectively). *Pax5* gene is flanked by *Melk* and *Zcchc7* genes. (**B**) Correlation between *Melk*, *Zcchc7* or *Ebf1* expression and *Pax5* expression. Quantitative PCR (QPCR) was performed at least as triplicate on Ba/F3, 70Z3, 38B9, 18.81, A20 and WEHI-231 cell lines and on B and T cells. Relative expressions (RQ) to *Abl1* expression are expressed as mean with error bars representing RQMIN and RQMAX and constitute the acceptable error level for a 95% confidence interval according to Student’s *t* test. The square of the Pearson correlation (r^2^) is indicated for each comparison.

The murine *Pax5* gene encompasses a region of 392 kb of chromosome 4 from the end of its upstream neighbor gene, *Melk*, to the start of its downstream neighbor gene, *Zcchc7* (Figure 1A). *Pax5* has a reverse orientation compared to its two neighbors, from telomere to centromere (Figure 1A). The human *PAX5* gene has a similar organization covering a slightly larger region of 444 kb on chromosome 9. In order to clarify the transcriptional activities within the *Pax5* locus, quantitative RT-PCR (QPCR) was performed to measure the overall expression of *Pax5* transcripts and its neighboring genes (*Melk* and *Zcchc7*), together with *Abl1* as a widely expressed control gene and *Ebf1* as a transcriptional target of Pax5. Their expression were measured in a series of murine B cell lines representing different stages of B cell differentiation (from the less to the most differentiated: Ba/F3, 70Z3, 38B9, 18.81, A20 and WEHI-231) along with murine primary tissues (T and B cells, Figure 1B). Since *Pax5* expression is regulated by Ebf1, *Pax5* expression is highly correlated to the expression of *Ebf1*, as expected (Pearson correlation, r^2^ = 0.85). In contrast, expression of *Pax5* is independent of the expression of its two neighboring genes, *Melk* and *Zcchc7* (Pearson correlation, r^2^ = 0.40 and r^2^ = 0.54 respectively), suggesting that the regulatory elements of *Pax5* are not shared by *Melk* and *Zcchc7*.

### *Pax5* isoforms are differentially expressed during B cell differentiation

**Figure 2:**
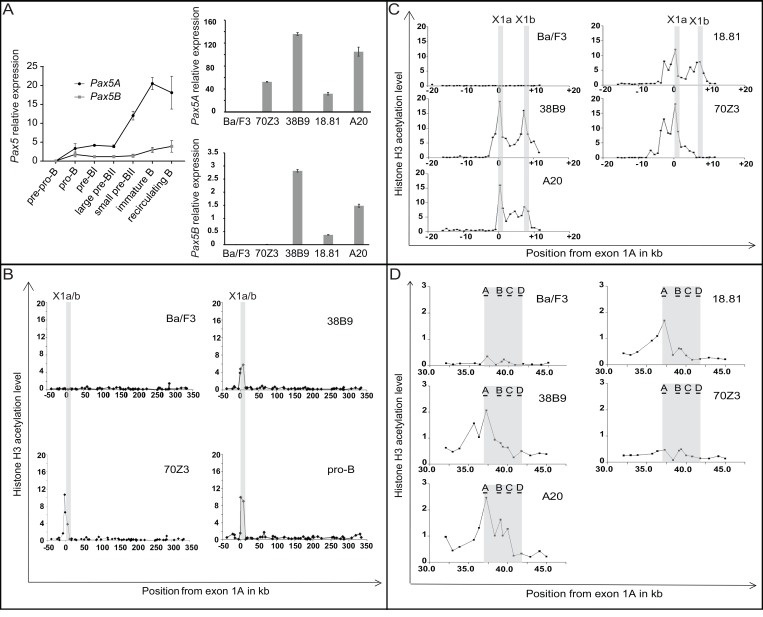
Correlation between *Pax5* isoforms expression and chromatin acetylation of the *Pax5* locus (**A**) QPCR of *Pax5A* and *Pax5B* isoforms on B cells during differentiation and in various cell lines. Expression of *Pax5* isoforms during medullar murine B cell differentiation was quantified on B cell subsets. Expression relative to *Abl1* are presented as mean and standard deviation (left panel); Expression of *Pax5A* (upper right panel) or *Pax5B* (lower right panel) on Ba/F3,70Z3, 38B9, 18.81, A20 cell lines and pro-B cells from murine bone marrow are expressed relative to *Gapdh* expression as mean and standard deviation. (**B**–**D**) Chromatin Immunoprecipitation (ChIP) analysis of *Pax5* locus on Ba/F3, 38B9, 70Z3 cell lines and murine pro-B sorted cells: QPCR was performed using primers covering the most conserved region (B) or located every 1 kb throughout promoter (C) or enhancer (D) regions. The increase over background was calculated by comparison with immunoprecipitates obtained using rabbit IgG antibody. Peaks of H3 acetylation are labeled according to their positions relative to the first nucleotide of *Pax5* exon lA.

Two major 5′ isoforms of *Pax5* are expressed during B cell differentiation. *Pax5A* expression is driven by the promoter 1A and *Pax5B* by the promoter 1B [using alternative first exons (1A and 1B respectively, Figure 1A)]. We detailed the expression pattern of the two *Pax5* isoforms during murine B cell differentiation using specific primers of these two isoforms on sorted B cell subsets from bone marrow. *Pax5B* has a low expression which does not vary during B cell differentiation. In contrast, *Pax5A* expression is strongly modulated during B cell differentiation with a higher expression in immature B cells (Figure 2A, left panel).

### Expression of *Pax5* isoforms is correlated with the histone H3 acetylation of their respective promoter

We defined the expression pattern of *Pax5* isoforms on B cell lines. The 38B9, 18.81, A20 cell lines expressed both *Pax5* isoforms. The early blocked 70Z3 B cell line expressed only *Pax5A* isoform. The Ba/F3 cell line did not express any of the two isoforms (Figure 2A, right panel).

Using the fact that B cell lines differentially expressed *Pax5* isoforms, we detailed the transcriptional regulation within the 392 kb genomic region based on the acetylation of histone H3, a major mark of active regulatory elements [17] at low resolution over the 50 most conserved regions in vertebrates. We observed a peak of acetylation covering *Pax5* promoters A and B in pro-B cells and the B cell lines expressing at least *Pax5A* in contrast to Ba/F3 (Figure 2B). We increased the resolution of the ChIP analysis to 1 kb over this region on five B cell lines (Figure 2C). The three cell lines expressing both *Pax5* isoforms (38B9, 18.81 and A20) exhibited two acetylation peaks located upstream of exon 1A (defined as the location origin, 0 kb) and 1B (+7 kb). 70Z3 expressing only *Pax5A* isoform had peaks at 0 kb and -4kb, but none at the +7 kb while Ba/F3 displayed no acetylation peak (Figure 2C).

Decker *et al.* identified an enhancer region located in the intron 5–6 of *Pax5* [24]. We analyzed the acetylation status of this region and showed that the acetylation is weak compared to the promoter regions. Interestingly, only the cell lines expressing both isoforms have an H3 acetylation mark on the enhancer region (Figure 2D).

### Both Pax5 isoforms restore early B cell differentiation

**Figure 3:**
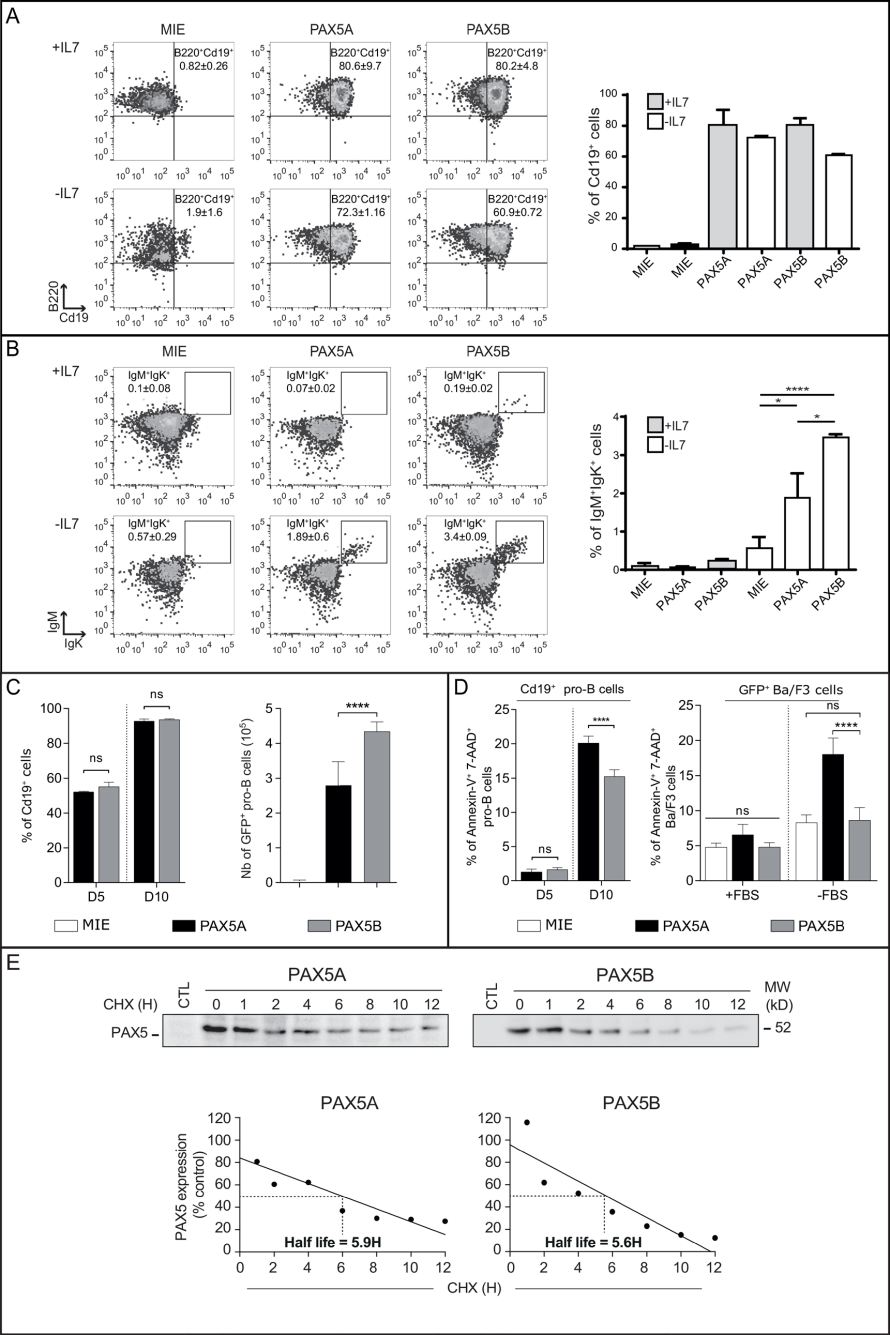
Both PAX5A and PAX5B can restore B cell differentiation program in complemented *Pax5*^*-/-*^ pro-B cells (**A**–**B**) Representative FACS analysis and statistics of differentiation of three independent infections of *Pax5*^-/-^ pro-B cells. Cells were gated on FSC/SSC criteria, 7-AAD negative population (living cells) and eGFP-positive cells (infected cells) (not shown). Gated cells were analyzed for (A) the presence of B220 and Cd19 (the percentage of Cd19^+^ cells is indicated on the right panel graph) or (B) IgM and Igk membrane markers (the percentage of IgM^+^/Igk^+^ cells is indicated on the right panel graph) in two different conditions: with (+IL7, upper line) or without IL7 (-IL7, lower line) for three days. The first column show *Pax5*^-/-^ cells infected with the eGFP-expressing vector (MIE), the second column with *PAX5A* retroviral construct and the third column with the *PAX5B* retroviral construct. The mean and the standard deviation of percentage of each population compared to the parental gate are indicated, ^*^p < 0.05, ^****^ *p* < 0.001. (**C**) Mean and standard deviation of percentage of Cd19^+^ cells (left panel); Absolute number of GFP^+^ *Pax5*^-/-^ pro-B complemented with eGFP (MIE), PAX5A or PAX5B after 3 days of culture for three independent infections, ^****^p < 0.001 (right panel). (**D**) Percentage of 7-AAD^pos^ AnnexinV^pos^ cells after 5 or 10 days in presence of IL7 (left panel) for three independent infections with *PAX5A*- or *PAX5B*-expressing vectors. ^****^p < 0.001. (**E**) Quantification of PAX5A and PAX5B proteins by Western-blot using an anti-Pax5 antibody after cycloheximide treatment for the indicated period of time on Ba/F3 infected cells demonstrating a similar half-life.

We took advantage of the *Pax5*^-/-^ cells which do not express any of the two isoforms and are subsequently blocked at a pro-B cell stage but still proliferating in presence of IL7. *PAX5* isoforms were individually transduced in *Pax5*^-/-^ pro-B cells and assessed for their ability to reinitiate B cell differentiation, using the expression of *Cd19*, a specific target of PAX5, and the appearance of IgM^pos^IgΚ^pos^ B cells as hallmarks of B cell progression. Cells infected with the empty vector (MIE, only expressing eGFP) did not induce expression of *Cd19*. In contrast, both isoforms induced the expression of surface Cd19 in presence or absence of IL7 (Figure 3A, respectively upper and lower panels). Furthermore, both isoforms were also able to initiate the expression of IgM and IgΚ at the cell surface after removal of IL7 (Figure 3B, lower panel).

Transduced *Pax5*^-/-^ cells were seeded at the same concentration and cultured for 3 days in presence of IL7. Although the percentage of Cd19^pos^ cells is the same after transduction with *PAX5A* or *PAX5B* (Figure 3C, left panel), the absolute number of pro-B cells transduced by *PAX5* isoforms was increased compared to the MIE with the most potent proliferation being driven by PAX5B (Figure 3C, right panel). Furthermore, after 10 days of culture in presence of IL7, *Pax5*^*-/-*^ pro-B cells complemented with PAX5B have a quarter less in spontaneous cell death rate compared with PAX5A (Figure 3D, left panel). This difference of cell survival may explain the highest percentage of IgM^pos^IgΚ^pos^ B cells after IL7 withdrawal with PAX5B complementation (Figure 3B).

We also stably transduced Ba/F3 cells with either MIE, *PAX5A* or *PAX5B* and stressed the cells by serum deprivation for 6 hours. Ba/F3 cells are less sensitive to serum deprivation when they express PAX5B compared to PAX5A whereas no difference was observed in 10% serum condition (Figure 3D, right panel). This difference is not linked to a difference of PAX5 isoform stability since half-life of the two proteins are comparable (Figure 3E). Consequently, the alternative use of *PAX5* exon 1 has no qualitative effect on early B cell differentiation but may have an impact on cell death.

### Both *Pax5* isoforms induce a B cell specific program

**Figure 4:**
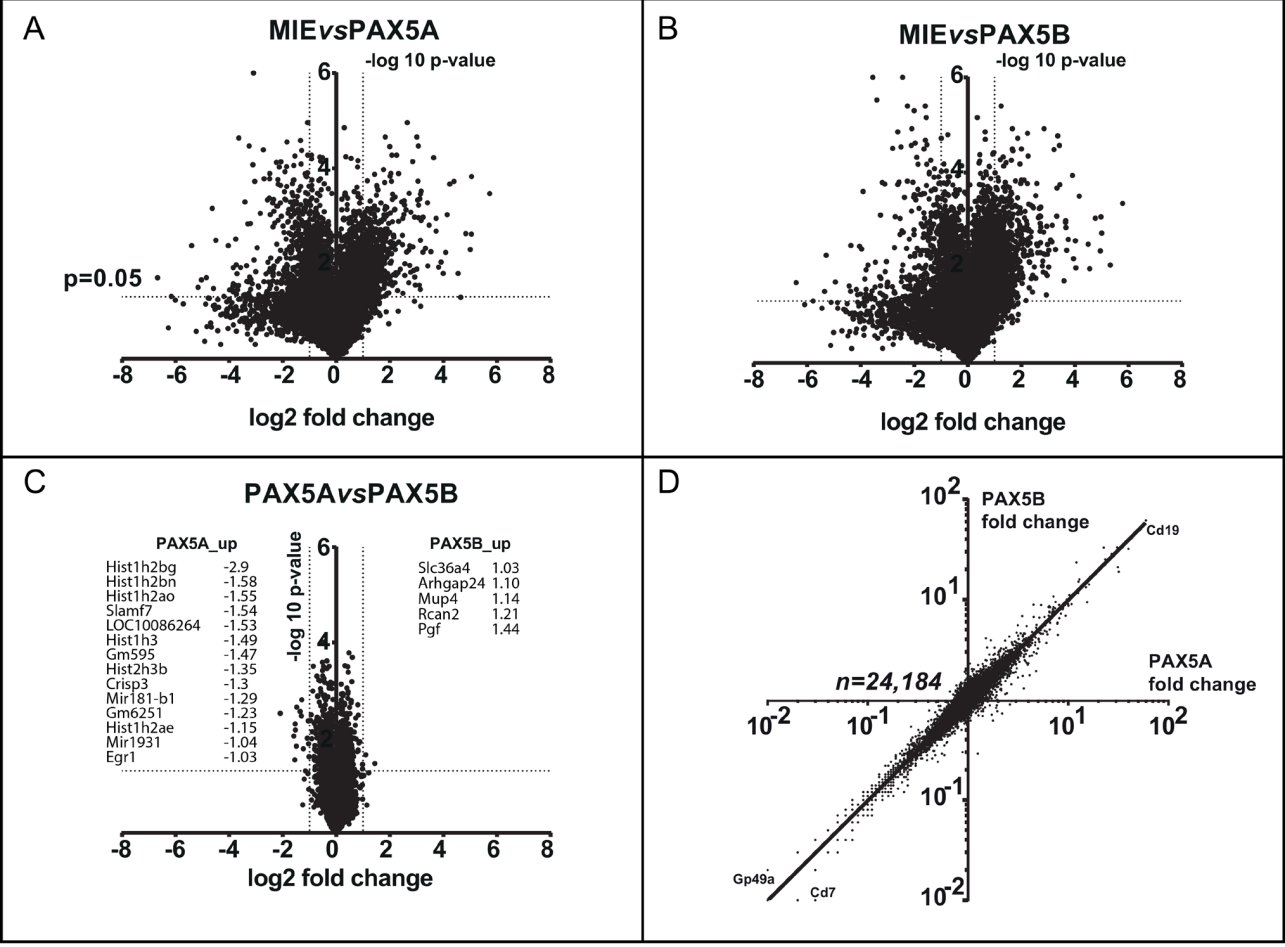
PAX5A and PAX5B drive a comparable transcriptomic early B cell program (**A**–**C**) Volcano plots presenting the fold change in log2 on x axis and the log10 *p*-value of the false discovery rate permutation analysis on y axis of the transcriptome comparing eGFP expressing vector (MIE) to PAX5A (A), MIE to PAX5B (B) and PAX5A to PAX5B (C) showing the similar effect of PAX5A and PAX5B on early B cell differentiation restoration. For C, gene with a difference of expression >2 fold between PAX5A and PAX5B are listed. (**D**) Scatter graph with gene expression fold changes between PAX5A and MIE on x-axis and PAX5B and MIE on y-axis. The regression line shows the relationship between PAX5A and PAX5B impact on target gene expression. As example, *Cd19* up-regulated gene and *Gp49a* and *Cd7* down-regulated genes are indicated.

After sorting transduced *Pax5*^*-/-*^ pro-B cells, their respective transcriptomes were analyzed (Figure 4A–4C). Both PAX5A and PAX5B enhance the expression of B cell specific genes such as *Cd19* and repress the expression of non-B cell specific markers such as *Cd7* (a T cell marker) or *Gp49a* (a myeloid marker) (Figure 4D). Furthermore, out of the 24,184 genes, no expression differs more than 5-fold between PAX5A and PAX5B target genes (Figure 4C). By performing a Gene Ontology (GO) analysis [18, 19] on the genes whose expression is the most discriminant, we found a significant enrichment in genes implicated in the regulation of apoptosis (Supplementary Figure 1A, 1B). These data strongly suggest that the two PAX5 isoforms globally induce B cell differentiation by activating the same targets during early B cell differentiation and differences in their target gene expression may explain the differences observed in cell death rate and cell growth (Figure 3C and 3D).

### Pax5 isoforms have a distinct pattern of expression during late B cell differentiation

**Figure 5:**
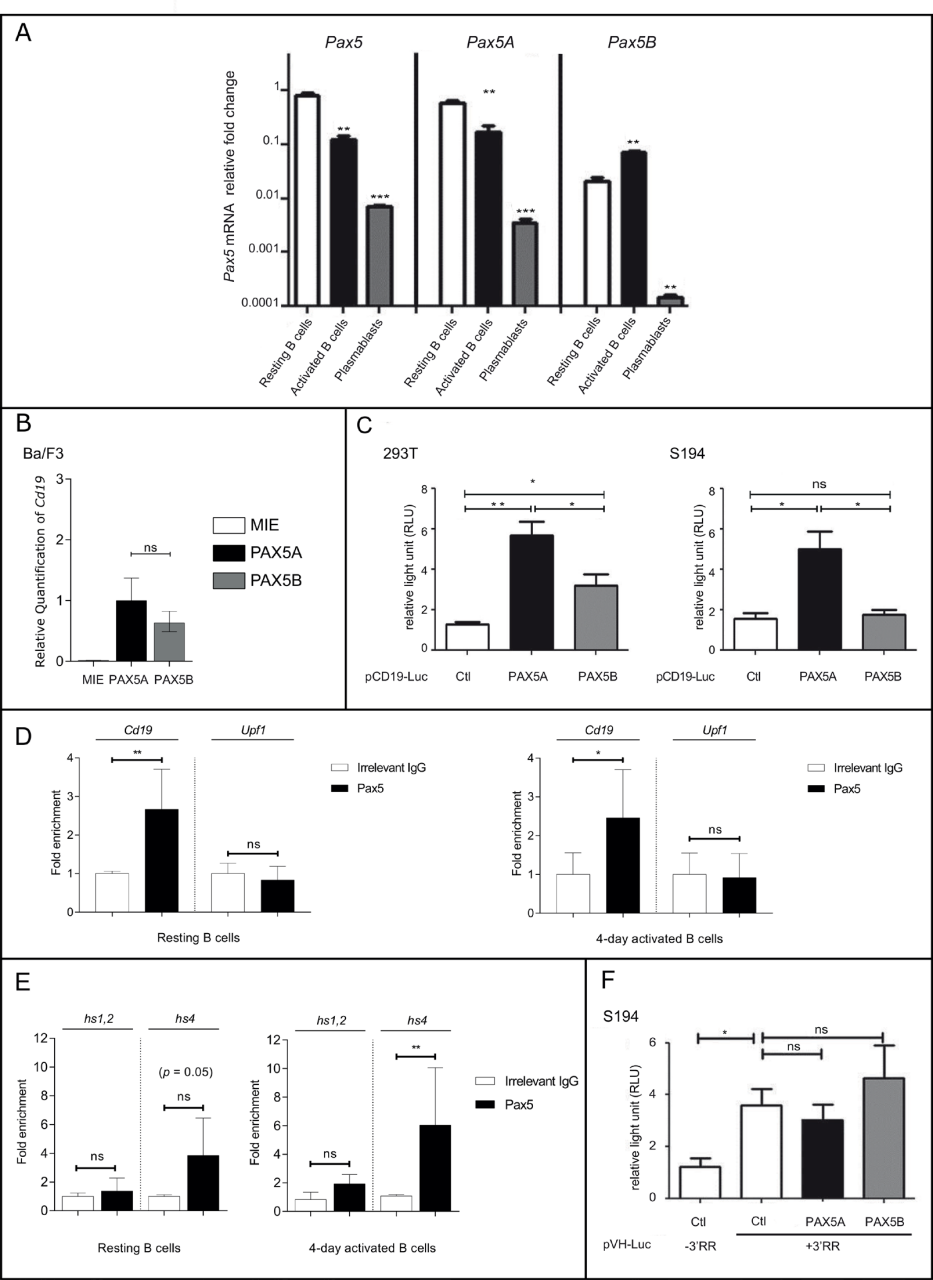
Pax5A and Pax5B show different activities during late B cell stages (**A**) Expression by QPCR of total *Pax5* or specific *Pax5* isoforms in resting murine B cells, activated B cells and plasmablasts. Results are expressed as log scale with mean ± SEM of independent experiments. (**B**) Relative quantification of *Cd19* transactivation in Ba/F3 after infection with either eGFP-expressing retrovirus (MIE) or MIE containing *PAX5A* or *PAX5B* (**C**) PAX5A or PAX5B trans-activity was determined using a Luciferase reporter (pCD19-Luc) assay in 293T cells (left panel) or S194 cells (right panel). Results are expressed as mean ± SEM of independent experiments. (**D**) ChIP was performed on *Cd19* promoter (pCD19) and Upf1 intron 9 (as a PAX5 non-target) and using an irrelevant IgG or an anti-PAX5 antibody. Results are expressed as mean of fold change compared to IgG condition ± SEM of independent experiments. (**E**) ChIP was performed on the hypersensitive sites (hs)1,2 and hs4 elements of the 3′RR in resting splenic B cells or activated B cells (left and right panel respectively) using an irrelevant IgG or a anti-PAX5 antibody. Results are expressed as mean ± SEM of independent experiments. (**F**) A *Luciferase* expression B cell specific vector (pVH-luc) containing the IGH 3′RR minilocus (+3′RR) or not (–3′RR) was used in a reporter assay to determine the regulatory function of PAX5A and PAX5B in S194 cells. Results are expressed as mean of fold change compared to IgG condition ± SEM of independent experiments. Ctl: negative control, ^*^p < 0.05; ^**^p < 0.01; ^***^p < 0,001 (unpaired two-tailed *t* test).

We also investigated the functional specificities of *PAX5* isoforms during late B cell differentiation. We quantified *Pax5* isoforms in murine sorted splenic resting B cells (Cd43^neg^ B220^pos^), *in vitro* LPS-activated B cells (B220^pos^ Cd138^neg^) and plasmablasts (B220^pos^ Cd138^pos^). Overall, *Pax5* expression is decreased after B cell activation (Figure 5A). In details, *Pax5A* was the most expressed isoform and downregulated in activated B cells and plasmablasts. In contrast, *Pax5B* was significantly upregulated in activated B cells and almost undetectable in plasmablasts. Therefore, the expression patterns of the two isoforms are distinct during late B cell differentiation.

### Pax5B is inactive in plasma cells

The differential regulation of *Pax5* isoforms at late B cell differentiation stages may reflect different properties of these isoforms. To assess this possibility, we evaluated the transactivation of *Cd19* at an early B cell stage using transduced Ba/F3; both PAX5A and PAX5B can efficiently transactivate *Cd19* (Figure 5B). We also evaluated the transactivation potential of PAX5A and PAX5B at later stage of B cell differentiation using S194, a plasmacytoma cell line, or in a non-lymphoid context using 293T, a human kidney cell line, using a luciferase reporter system containing the *Cd19* promoter region. Compared to AID, a negative control, both PAX5A and PAX5B activate the *Cd19* promoter (Figure 5C). The activity of PAX5B is significantly lower than PAX5A. In S194, only PAX5A displays transcriptional activity suggesting that PAX5B transactivation capacity on *Cd19* promoter is altered in those cells (Figure 5C). To characterize the binding of Pax5 on the *Cd19* promoter at late stages of the B cell differentiation, we precipitated chromatin (ChIP) using an anti-Pax5 antibody in splenic B cells (Figure 5D). We detected a specific binding of Pax5 to the *Cd19* promoter sequence in resting B cells compared to the amplification of an irrelevant sequence (*Upf1* intronic sequence) devoid of Pax5 binding sites (Figure 5D, left panel). After 4 days of LPS stimulation, we could detect the same enrichment of Pax5 on *Cd19* promoter (Figure 5D, right panel).

### Neither PAX5A nor PAX5B are able to silence the 3′RR in plasma cells

The 3′RR is an important region regulating the expression of the heavy immunoglobulin chain at late B cell differentiation stages. It has been reported that Pax5 could bind hypersensitive sites (hs)1,2 (3′αE) and hs4 elements ([20, 21]). A *Pax5*-specific ChIP using an antibody that does not discriminate between the two isoforms, identified a 3.8-fold enrichment at the hs4 element site in resting B cells (Figure 5E, left panel). Upon activation, Pax5 is enriched both at the hs4 and to a much lesser extend to hs1,2 sites (6-fold enrichment for hs4 and a 2.4-fold for hs1,2; Figure 5E, right panel).

We then analyzed whether both *PAX5* isoforms have a similar transactivation activity on the 3′RR in S194 plasma cells (Figure 5F). *PAX5* isoforms are physiologically downregulated in plasma cells, in contrast the 3′RR activity is increased. We evaluated the effect of the *PAX5* isoforms on the 3′RR activity using a reporter assay depending on the *VH* promoter (pVH) active in the B cell lineage. Its transcription level is upregulated by the addition of a synthetic 3′RR containing the four enhancers in their palindromic structure (hs3a, hs1.2, hs3b, hs4). We did not observe any significant change upon the addition of both *PAX5* isoforms, suggesting that 3′RR is insensitive to Pax5 (Figure 5F) despite the accumulation of Pax5 at the 3′RR after activation of B cells (Figure 5E, right panel).

## DISCUSSION

*PAX5* is a major regulator of B cell differentiation promoting the expression of B cell specific genes and repressing commitment to other cell lineages. Consequently, to allow an efficient B cell differentiation, the expression of *PAX5* has to be tightly controlled. An alteration of *PAX5* dosage induced B cell malignancies: its overexpression, secondary to the *IGH-PAX5* rearrangement, is associated with B cell lymphomas [16] and loss of heterozygosity is associated with B cell progenitor acute lymphoblastic leukemia [22, 23]. The regulation of *PAX5* expression, in vertebrates, is complex with two 5′ isoforms, *PAX5A* and *PAX5B*, initiated from two different promoters. These isoforms differ only by the sequence of their first coding exon encoding no specific protein motif. PAX5B isoform is related to PAX2, since the sequence encoded by their first exon are 65% homologous and is present as early as in the Fugu (Table 1). The *PAX5A* isoform appears later, with mammals and by extension B cells.

**Table 1:** Comparative analysis of exon 1A and exon 1B coding sequences during evolution

Species	PAX5 coding exon 1A	PAX5 coding exon 1B
Human	MDLEKNYPTPRTSRT	MEIHCKHDPFASMH
Chimpanzee	MDLEKNYPTPRTSRT	MEIHCKHDPFASMH
Mouse	MDLEKNYPTPRTIRT	MEIHCKHDPFASMH
Rat	MDLEKNYPTPRTIRT	MEIHCKHDPFASMH
Rabbit	MDLEKNYPTPRTGRT	MEIHCKHDPFASMH
Dog	MDLEKNYPTPRSGRT	MEIHCKHDPFASMH
Pig	MDLEKNYPTPRTGRT	MEIHCKHDPFASMH
Cow	MDLEKNYPTPRTGRT	MEIHCKHDPFASMH
Xenopus	No hits found	MEIHCKHDPFAAMH
Zebrafish	No hits found	MEIHCKHDPFAAMH
Fugu	No hits found	MEIHCKHDPFAAMH
C. elegans	No hits found	No hits found
Fruitfly	No hits found	No hits found
Lamprey	No hits found	No hits found

The table shows the alignment of PAX5 exon 1A and exon 1B primary sequence in different species. The discrepancies between species are boxed.

Very little is known about the differential regulation of these two isoforms and their functional differences. The aim of this study was to determine if the presence of these two isoforms translate a functional difference or are merely a differential regulation of expression. In B cells, *PAX5* isoforms are differentially expressed suggesting that different sets of transcription factors enhance or repress the activity of these promoters. ChIP analyses on cell lines and pro-B cells confirm a correlation of histone H3 acetylation on both promoter regions and expression of the two isoforms. Interestingly, we also detected histone H3 acetylation on the enhancer region [24] only when the two isoforms are expressed.

Functionally, PAX5A and PAX5B can act similarly in early B cell differentiation. Both are able to complement the invalidation of *Pax5* in *Pax5*^-/-^ cells and resume *ex vivo* the B cell differentiation until the Cd19^pos^/mIgΚ^pos^ stage demonstrating a similar capacity to transactivate or repress target genes during this process. This is shown by the transcriptome since no gene expression differs more than five times in *Pax5*^-/-^ complemented by PAX5A or PAX5B. Nonetheless, we detected differences between the two isoforms. PAX5B confers a growth advantage of the rescued *Pax5*^-/-^ cells compared to PAX5A-rescued cells. The physiological up-regulation of *Pax5B* during B cell activation may prevent apoptosis of activated B cells since suppression of PAX5B increases susceptibility of human acute lymphocytic leukemia REH cells to apoptotic death [25]. Interestingly GO term analysis on discriminant genes for which the expression is at least 1.5 fold higher for PAX5A or PAX5B condition shows that they are involved in cell death pathways.

As described by Revilla-i-Domingo *et al.*, although Pax5 interacts with a similarly high number of genomic binding sites defining 8,000 target genes in pro-B and mature B cells, only 13 target genes are commonly activated and 18 target genes are commonly repressed in pro-B and mature B cells (cited from [26]) suggesting that Pax5A and Pax5B could have different transcriptional activities in early and late B cell differentiation stages.

At later stages of B cell differentiation, we observed that *Pax5B* and *Pax5A* are expressed simultaneously in primary B cells at different levels. After B cell activation, they exhibit a distinct pattern of expression: *Pax5A* decreases in activated B cells and plasmablasts while *Pax5B* increases in activated B cells and is virtually absent in plasmablasts.

PAX5A is able to transactivate *Cd19* promoter in both early (Ba/F3) and late B cells (S194) and in a non-lymphoid 293T whereas PAX5B is able to transactivate *Cd19* only at an early stage of B cell differentiation and in a non-lymphoid context. These results point out that PAX5B isoform cannot transactivate the *Cd19* promoter in a late lymphoid context (S194) or may function in a different context as transcriptional repressor as proposed by Robichaud *et al.* The authors have shown that PAX5B negatively regulates *CD19* expression in REH cell line since the specific suppression of *PAX5B* expression using ribozymes leads to an increase of *CD19* expression [25].

PAX5 has been described as a negative regulator of the 3′RR activity. Nonetheless, we observed an increased binding of PAX5 to the 3′RR upon B cell activation. The accumulation of PAX5 binding on the 3′RR in activated B cells does not seem to reflect a role of PAX5 in the 3′RR activity regulation but rather a function in chromatin remodeling permitting accessibility to the *IGH* locus. Recently, it has been shown that PAX5 recruits PTIP, a molecule involved in class switch recombination [27, 28] which induces H3K4me3 and stabilizes the PAX5 binding on the *IGH* locus [28]. Alternatively, this could be explained by the fact that the activation of B cells results in the induction or increase of the expression of other factors counteracting PAX5 activity.

PAX5 isoforms are physiologically downregulated in plasma cells in contrast to the 3′RR activity which is strongly increased. We investigated whether these isoforms could modify the 3′RR activity. Our data showed that 3′RR is insensitive to a PAX5A or PAX5B regulation in the S194 plasmacytoma cell line, in agreement to the observation that an hs1,2-dependent transgene is not inhibited whereas Pax5 expression is still detected in activated B cells [29]. Consequently, repression of the 3′RR activity by Pax5 [20, 30, 31] is likely more complex.

Our results reveal only discrete differences of action between PAX5A and PAX5B functions and demonstrate that these two isoforms may be interchangeable in early B cell differentiation. Their very tight regulation may reflect different binding of alternative transcription factors according to the cellular context (testis, brain, B cells) leading to a fine control of the *Pax5* dosage which is required for a zero-default B cell differentiation.

## MATERIALS AND METHODS

### Cell line culture

In this study, we used several B cell lines showing characteristics of various stages of B cell differentiation: Ba/F3 are pro-B cells (DSMZ ACC 300) [32], 38B9 are pro-B cells [33, 34], 70Z/3 are pre-B lymphoblast (ATCC^®^ TIB-158™) [35], A20 is a BALB/c B cell lymphoma line derived from a spontaneous reticulum cell neoplasm found in an old BALB/cAnN mouse (ATCC^®^ TIB-208™) [36], 18.81 is a Abelson-virus-transformed mouse lymphoid cell line with pre-B cell characteristic [37], WEHI-231 are immature B cells (ATCC^®^ CRL-1702™) [38], S194 are mouse myeloma B cells, this line was derived from an IgA secreting mineral oil induced BALB/c myeloma. (ATCC^®^ TIB-19™) [39]. Murine B cell lines S194, 38B9, A20, 18.81, and WEHI-231 were cultured in RPMI (Gibco), 10% FBS (fetal bovine serum, PAN Biotech), 2 mM L-glutamine, and 1 mM sodium pyruvate (Invitrogen). The same medium supplemented with 0.05 mM β-mercaptoethanol or 10 ng/ml recombinant murine IL3 (Peprotech) was used respectively to culture the murine cell line 70Z3 or Ba/F3. The Phoenix retrovirus producer line (Orbigen) was cultured in high glucose Dulbecco’s Modified Eagle’s Medium (DMEM; Gibco), 10% FBS (PAN Biotech). Murine OP9 bone marrow stromal cells were cultured in MEMα (Gibco), 20% FBS (PAN Biotech) when in monoculture. All the cells were cultured in presence of penicillin (100U/ml) and streptomycin (100U/ml) at 37° C, in 5% CO_2_ atmosphere.

### Mice

*Pax5*± mice were kindly provided by Pr M. Busslinger [6]. *Pax5*^-/-^ and *Pax5*^*+/+*^ (wild-type) mice were obtained by breeding two Pax5± mice. All mice were maintained on the C57BL/6 genetic background. The Pax5 genotype was determined using REDExtract-N-Amp Tissue PCR Kit according to the manufacturer’s instructions (Sigma-Aldrich) with the following oligonucleotides: 5′-AGG ACA TGG AGG AGT GAA TCA G-3′ (primer1); 5′-ACC CGA AGC TGC CTG GAG-3′ (primer2); and 5′-AGG CGA TTA AGT TGG GTA ACG-3′ (primer 3). All animal experiments were carried out according to valid project licenses, which were approved and regularly controlled by French Authorities.

### Purification and culture of B cell subsets

*Pax5*-deficient pro-B cells were harvested from *Pax5*^-/-^ embryonic liver at E17.5. Cells were amplified on γ-irradiated (30 Gy) OP9-derived stromal cells in Iscove’s Modified Dulbecco’s Medium (IMDM; Gibco), supplemented with 5% FBS (Stemcell technologies), 100 U/ml murine IL7 (Peprotech), 0.05 mM β-mercaptoethanol (Sigma-Aldrich), 2 mM L-glutamine (Invitrogen), penicillin (100 U/ml) and streptomycin (100 U/ml). Wild-type pro-B cells were flushed from bone marrow. After red blood cell lysis, B cell fraction was enriched by B220 magnetics microbead sorting (Myltenyi Biotech). B cell fraction was subsequently incubated with B220, Kit (Cd117) and Igk antibodies and pro-B cells (B220^pos^ kit^pos^ and Igκ^neg^) were sorted using a FACS-Vantage sorter (BD Biosciences). Splenic resting B cells were purified by negative selection using anti-CD43 microbeads (Miltenyi Biotech) and stimulated (0.5 × 10^6^ cells/ml) by LPS (20 µg/ml; Sigma) for 2 to 4 days in RPMI supplemented with 10% FBS, sodium pyruvate, non-essential amino acids, β-mercaptoethanol, penicillin (100 U/ml) and streptomycin (100 U/ml) (Invitrogen).

B cell subsets were sorted on a MoFlo Astrios sorter (Beckman Coulter) after bone marrow flush of C57Bl/6 8 to 12 week-old mice as follows: pre-pro-B (B220^+^Cd19^-^), pro-B (B220^+^Cd19^+^IgL^-^IgD^-^Cd117^+^BP1^-^), pre-BI (B220^+^Cd19^+^IgL^-^IgD^-^Cd117^+^BP1^+^), large pre-BII (B220^+^Cd19^+^IgL^-^IgD^-^Cd117^+^, large FSC), small pre-BII (B220^+^Cd19^+^IgL^-^IgD^-^Cd117^+^, small FSC), immature B (B220^+^Cd19^+^IgL^+^IgD^-^), recirculating B (B220^+^Cd19^+^IgL^+^IgD^+^) and RNA extraction were performed on fresh samples.

For LPS stimulation B cells (B220^pos^ Cd138^neg^) and plasmablasts (B220^pos^ Cd138^pos^) were sorted 3 to 4 days after stimulation.

### Quantitative RT-PCR

RNA was isolated using the AllPrep™ DNA/RNA Micro Kit (QIAGEN) and cDNA was synthesized using SuperScript^®^ VILO™ cDNA Synthesis Kit (Invitrogen™) according to the manufacturer’s instructions. Quantitative PCR were performed on a LightCycler^®^480 II System (Roche). Quantitative SYBR Green PCR was performed to quantify relative mouse Cd19 cDNA expression in transduced Ba/F3 using LightCycler^®^480 SYBR Green I Master (Roche Diagnostics GmbH) according to the manufacturer’s instructions, and primers 10 µM primers for *Cd19* (forward: 5′-AAGGAACAGGTCCTCTGGGA-3′ and reverse: 5′-GATTCAAACTGCTCCCCCGA-3′) and 10 µM primers for *Abl1* (forward: 5′-GGAGAAGGTCTACGAGCTCAT-3′ and reverse: 5′-ATCTGAGATACTGGATTCCTGGA-3′). Relative gene expression in cell lines or sorted bone marrow B cells was quantified using LightCycler^®^480 Probes Master (Roche Diagnostics GmbH) and the following TaqMan gene expression assays from Applied Biosystems™ Life Technologies: Mm00802038_m1 for *Abl1*, Mm00487680_m1 for *Melk*, Mm00435501_m1 for *Pax5* (both isoforms), Mm00621713_m1 for *Zcchc7*, Mm00395519_m1 for *Ebf1*, Mm00435494 for specific expression of *Pax5A*. Primers/TaqMan probe mix specific for PAX5B transcripts was synthetized by Applied Biosystems (5′FAM-ATG CAT AGA CAT GGA GGA GTG AAT CAG CTT G-Tamra3′ as probe; 5′-CAC TGT AAG CAC GAC CCG TT-3′ as forward primer; 5′-AGT GGC CGT CCA TTC ACA AA-3′ as reverse primer). All QPCR programs were carried out as follows in a 20 µl volume: 5 min at 95° C, followed by 45 cycles of 10 s at 95° C, 10 s at annealing temperature of 60° C and 10 s at 72° C. All signals were quantified using either the ΔCt method or the Relative Quantification (ΔΔCt) study both including a normalization to the ΔCt values of *Abl1* gene expression levels. Data were analyzed using the LC480 software (Roche Diagnostics) followed by statistical analyses.

### ChIP experiment

CHromatin ImmunoPrecipitation (ChIP) assays were performed essentially as described previously [40] using anti-acetylated histone H3 or nonspecific rabbit immunoglobulin G (IgG) (06-599 and 12-370, respectively; Upstate Biotechnology, Lake Placid, NY). Oligonucleotide sequences are available on Supplementary Table 1. Murine splenic B cells were cultured (10^6^ cells/ml) in presence of LPS (40 µg/ml) for 4 days. At day 3, dead cells were eliminated by Ficoll centrifugation (Lympholyte-Mammal, Cedarlane) and living cells were stimulated for an additional 24 h period. Chromatin preparations were performed on 5 × 10^7^ to 10^8^ LPS-stimulated B cells using standard fixation and sonication methods as described [41]. In all cases, 200 ng of chromatin was immunoprecipitated (ON, 4° C) with 1µg of anti-PAX5 mAb (mouse anti-Pax-5 mAb (A-11), Santa Cruz Biotechnology) or an irrelevant IgG antibody (purified mouse unlabeled IgG1, SouthernBiotech, ref.0102-01). Oligonucleotides used are given in Supplementary Table 1.

### Retroviral particles production

Full-length human *PAX5A* and *PAX5B* cDNA were amplified, sequenced and inserted into the retroviral vector pMSCV-IRES-eGFP (MIE), a bicistronic vector allowing the expression of eGFP under the control of the long terminal repeat (LTR) promoter. Retroviral supernatants were produced by Phoenix retrovirus producer line (Orbigen) using Lipofectamine 2000 (Invitrogen) reagent for transfection and proceeding according to the manufacturer’s instructions. After overnight incubation of the transfection mix, cells were treated with 10 mM sodium butyrate (Millipore) and washed with PBS before pro-B cell medium was added. Viral supernatants were harvested after 24 h of incubation at 32° C, passed through a 0.45 µm filter, aliquoted and frozen at –80° C.

### Pro-B culture, infection and differentiation

The *in vitro* pro-B cell differentiation/proliferation protocol used was adapted from Rolink *et al.* [42]. The pro-B *Pax5*^-/-^ cells were isolated from *Pax5*^-/-^ embryo fetal liver and cocultivated with irradiated OP9 cells in 24-well plates in Iscove’s Modified Dulbecco’s Medium (IMDM; Gibco), supplemented with 5% FBS (Stemcell Technologies), 2 ng/ml murine IL7 (Peprotech), 0.05 mM β-mercaptoethanol (Sigma-Aldrich), 2 mM L-glutamine (Invitrogen), penicillin (100 U/ml) and streptomycin (100 U/ml). Experiments were performed after an initial pro-B cell expansion on irradiated OP9 cells in the presence of 2 ng/ml IL7. Every 3 days, pro-B cells were harvested and propagated on fresh OP9 stromal cells.

The pro-B cells, cultured on irradiated OP9 cells in the presence of 2 ng/ml of IL7, are infected during exponential growth. Retroviral supernatant was diluted 1:3, supplemented with 4 µg/ml polybrene (Sigma) and 2 ng/ml of IL7 (Peprotech); spinoculation was performed at 1000 × g at 32° C for 90 min. After centrifugation, 1ml of fresh medium supplemented with IL7 was added and the cells were incubated overnight at 37° C. After infected pro-B cell expansion for few days, cells were washed out of IL-7 and plated in IMDM, 5% FBS, 0.05 mM β-mercaptoethanol and 2 mM L-glutamine on irradiated OP9-derived stromal cells with or without IL7. At day 2.5, single-cell suspensions were stained using standard protocols for flow cytometry using the antibodies listed above. Dead cells were excluded from FACS analysis as 7-amino-actinomycin D (7-AAD; BD Biosciences #559763)) positive (BD Biosciences). Stained cells were analyzed with a FACS Calibur flow cytometer (BD). Data were analyzed with the BD FACSDiva™ software (BD Biosciences) or FlowJo (TreeStar, Ashland, OR). All antibodies are rat monoclonal antibodies from BD biosciences: anti-B220 (clone RA3-6B2) conjugated with pacific blue, anti-CD19 (clone 1D3) conjugated with Alexa Fluor 700, anti-mouse Igk light chain (clone 187.1) conjugated with Phycoerythrin-Cyanin7, anti-IgM (clone R6-60.2) conjugated with Peridinin chlorophyll protein-cyanin5.5.

Ba/F3 cells were similarly transduced with empty MIE, MIE-PAX5A or MIE-PAX5B and then GFP-positive cells were isolated by fluorescence-activated cell sorting (FACS) in MoFlo Astrios (Beckman Coulter).

### Cycloheximide treatment and Western blot

Stability assay was performed in the presence of cycloheximide (100 μg/mL, Sigma) for the indicated periods of time. The day before, 1.2 × 10^6^ transduced Ba/F3 cells were incubated overnight at 37° C, 5% CO_2_. At each time point, treated cells were washed once with ice-cold Phosphate buffer saline (PBS) and then cell pellets were lysed in 40 μl of lysis buffer composed of 150 mM NaCl, 1 mM EDTA, 50 mM Tris, 1% Triton and protease inhibitor cocktail (cOmplete™, Roche). Protein concentrations of cell lysates were determined using the BCA1-Kit for Protein Determination (Sigma-Aldrich) to load 30 µg in every well. Samples were subjected to 10% sodium dodecyl sulfate-polyacrylamide gel electrophoresis and transferred to PVDF membranes. Blots were blocked 1 hour in the presence of 5% milk and incubated with 0.3 μg/mL of primary antibody overnight at +4° C (mouse anti-Pax-5 mAb (A-11), Santa Cruz Biotechnology). After incubation with secondary antibodies conjugated to horseradish peroxidase (Cell Signaling Technology), the immunoreactive bands were visualized by using the enhanced chemiluminescence lighting system (Amersham™ ECL™ Prime Western Blotting Detection Reagent, GE Healthcare) and data was acquired with Chemi-Smart 5000 and Bio-1D software (Vilber Lourmat). Bands were quantified by using ImageJ software.

### Flow cytometry and cell growth

Transduced pro-B cells and Ba/F3 cells were stained with AlexaFluor700-conjugated anti-CD19 (1D3) antibody (BD Pharmingen). For cell-viability assays, cells were stained with Annexin-V-PE and 7-AAD (BD Biosciences #559763). Flow cytometric data were acquired on a Fortessa X20 or LSR II flow cytometer (BD Biosciences), and data were analyzed using FACSDiva (BD Biosciences) and FlowJo software (TreeStar).

### Transcriptomic analysis

After 5 days in culture with 2 ng/ml of IL7, infected cells were sorted according to their positivity for GFP fluorescence with a FACSAria II (BD Biosciences) cell sorter. Total RNA from GFP^pos^ sorted pro-B infected cells were extracted using the Trizol method according to manufacturer’s instruction (Invitrogen). RNA quality was assessed using the RNA 6000 Nano Assay on the Agilent 2400 Bioanalyser (Agilent Technologies, Massy, FRANCE). For each condition (PAX5A, PAX5B and MIE), three independent infection experiments were performed. RNA samples were purified and prepared according to the manufacturer’s protocol with the Affymetrix’s GeneChip Whole Transcript Sense Target Labeling Assay Kit (Affymetrix, UK) and hybridized on Affymetrix GeneChip Mouse Gene 2.0 ST arrays. Probe-signal intensities were normalized and summarized by the Robust Multiarray Average (RMA) method using Affymetrix’s Expression Console software.

The data discussed in this publication have been deposited in NCBI’s Gene Expression Omnibus [43] and are accessible through GEO Series accession number GSE104890 (https://www.ncbi.nlm.nih.gov/geo/query/acc.cgi?acc=GSE104890).

For the volcano plots, permutation analyses were performed with dChip software [44] allowing the calculation of false discovery rate *p*-value and the fold difference volcano plots were generated using GraphPad prism software.

The gene ontology analysis has been performed with the AutoCompare ZE software [45] using Gene Ontology database [18, 19], *p*-values have been calculated with the ZE test.

### Luciferase reporter assay

Experiments were performed by cell transfections with 2µg DNA at 1:7:1 (pcDNA3-PAX5 vector: reporter vector: GFP-CMV) vector ratios. GFP-CMV was used to evaluate transfection efficiency. The S194 line was cultured in RPMI as above and for each condition 2 × 10^6^ cells were electroporated according to the manufacturer’s instructions (Lonza). The 293T line was cultured in DMEM (with 10% FBS, sodium pyruvate, nonessential amino acids, penicillin and streptomycin) and subconfluent cells in 6-well dishes were transfected by lipofection with Exgen500 (Euromedex). Firefly luciferase-CD19-2(A ins) [46] was kindly provided by Pr M. Busslinger was used to measure PAX5 activity on the PAX5 binding site of the *Cd19* promoter. PAX5 activity on the 3′RR was evaluated with firefly luciferase-pV_H_ enhanced or not by the combination of core enhancers of 3′RR [47]. After 48 h transfection, cells were collected and split for GFP fluorescence analysis by flow cytometry and luminescence detection using the Luciferase Reporter Assay System and the 20/20^n^ Luminometer (Promega). For each sample, luminescence was normalized to the measured GFP fluorescence.

### Statistical analysis

Experiments have been performed at least as three independent conditions. All statistical analyses were performed using Excel (Microsoft) and GraphPad Prism software, version 7 (GraphPad Software). Statistical differences were determined using a 2-tailed Student’s *t* test or RM one-way ANOVA with Tukey’s correction or one-way ANOVA with Sidak’s correction for multiple comparisons or Pearson correlation, as mentioned in legends. All data are presented as mean ± SD or SEM. A *p*-value of less than 0.05 was considered statistically significant, the null hypothesis being rejected at the 0.05 level (^***^p < 0.0005, ^**^p < 0.005, ^*^p < 0.05).

## SUPPLEMENTARY MATERIALS FIGURE AND TABLE




