# Supplementary material for: PAX5A and PAX5B isoforms are both efficient to drive B cell differentiation
Source: Oncotarget. 2018 Aug 28;9(67):32841–54. doi: 10.18632/oncotarget.26003 (PMC6132355; doi:10.18632/oncotarget.26003)
Supplement: Supplementary file 1 [file oncotarget-09-32841-s001.pdf]

PAX5A and *PAX5B* isoforms are both efficient to drive B cell differentiation

SUPPLEMENTARY MATERIALS

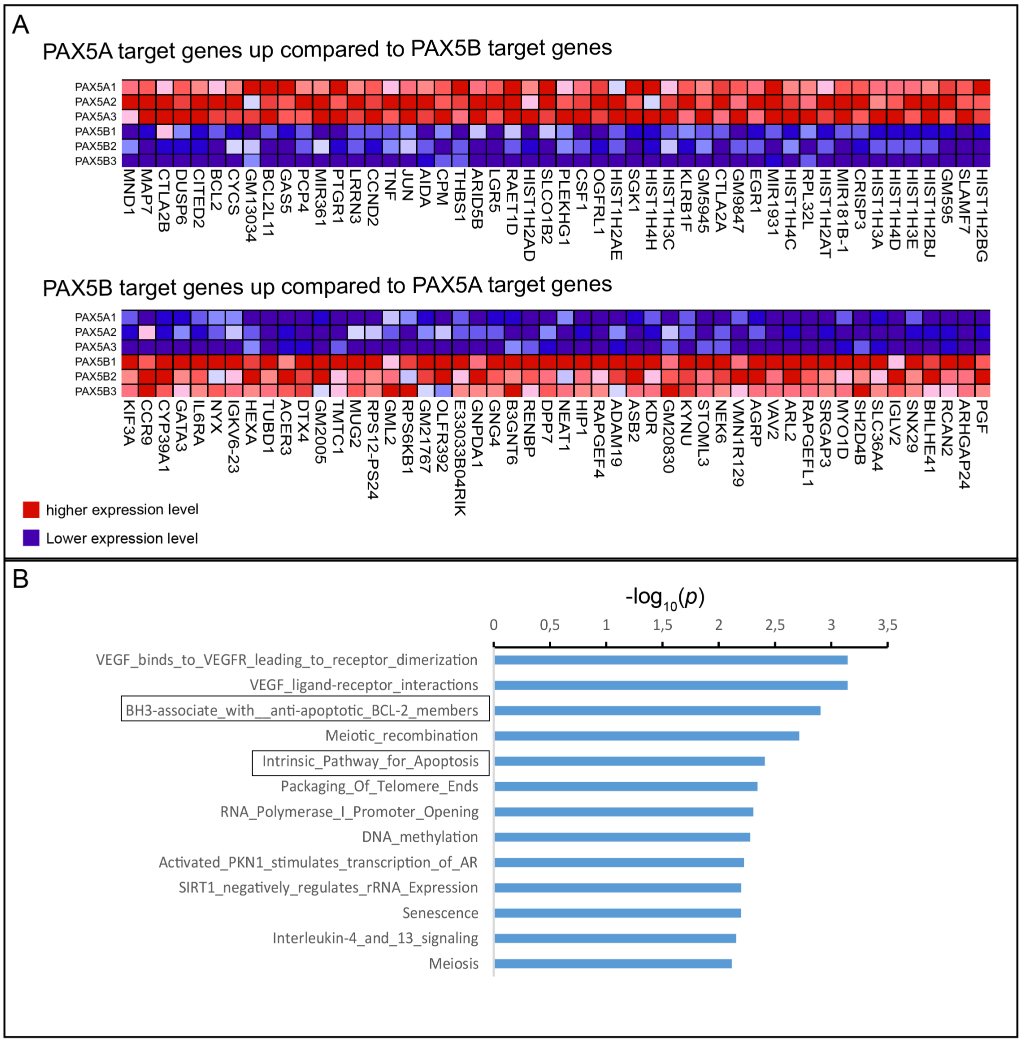

Supplementary Figure 1: (**A**) Heatmaps showing the 100 most differential genes expression using dChIP software ([44]): (upper panel) top fifty genes whose expression is higher for PAX5A (in red) compared to PAX5B (in blue) in *Pax5*$^{-/-}$ pro-B infected cells [PAX5A/PAX5B target gene expression ratio range (4.25 to 1.58)], (lower panel); top fifty genes whose expression is lower for PAX5A (in blue) compared to PAX5B (in red) in *Pax5*$^{-/-}$ pro-B infected cells [PAX5B/PAX5A target gene expression ratio range (2.72 to 1.49)]. (**B**) Gene ontology analysis of differentially expressed genes showing the 13 most significantly enriched biological pathways, –Log$_{10}$ (*p*) is indicated in the X axis.

Supplementary Table 1: Oligonucleotides used for murine ChIP analyses. See Supplementary_Table_1
