# Supplementary material for: PAX5A and PAX5B isoforms are both efficient to drive B cell differentiation
Source: Oncotarget. 2018 Aug 28;9(67):32841–54. doi: 10.18632/oncotarget.26003 (PMC6132355; doi:10.18632/oncotarget.26003)
Supplement: Supplementary file 2 [file oncotarget-09-32841-s002.docx]

Supplementary Table 1: Oligonucleotides used for murine ChIP analyses

| Forward primer | Sequence | Reverse primer | Sequence | Distance to ATG  of Pax5 exon 1A |
| --- | --- | --- | --- | --- |
| MP5_25-117F | TGCAATTTGAAGAGCTGCAGA | MP5_25-217R | TTCTTGGGAATTCTCAATGACATC | -38 064 |
| MP5_15-180F | CATGCTCTGACAACTTATTCTGCA | MP5_15-280R | TCGCAGGAACACATGAATAGCT | -22 357 |
| MP5_17-135R | GCCGGTGCTGAGGCTAACTA | MP5_17-13F | CCATTAGGAGACCAGTTCAGCAA | -17 727 |
| MmP5_361-524F | AAAGCGGGAGTCAGACGTGT | MmP5_361-625R | GCATGCAACTGGAGATGAGCT | -15763 |
| MP5_07-322F | ACCCGGCAATGCAGGTTTA | MP5_07-431R | TTTATCCAGAATAACCCGACATGA | -15 575 |
| MmP5_360-452F | CCGTGGTAGTCAATGTTGCCTAT | MmP5_360-553R | TTCAGCACTACTGTGGAACAATCA | -14691 |
| MmP5_359-637F | ATGGACATCCTGGCACCAAT | MmP5_359-738R | GGTCTGAAAGTCAGGTCTTGCA | -13876 |
| MmP5_358-365F | CATGACCTGGAAATGGTGACC | MmP5_358-467R | AACACGGCCTTGCTCTCCT | -12604 |
| MmP5_357-483F | CTGTCACCGTGTAGCCCCAT | MmP5_357-583R | AGCAAGACAAAAGCCAGGCA | -11722 |
| MmP5_356-719F | CTCAACACAAGCGTGGAGGTT | MmP5_356-822R | CTGCCAGTCAGGCTCTTGG | -10958 |
| MmP5_355-481F | GATGGACGCAGCGTTGCT | MmP5_355-598R | TCGTTGATGTGCTATTGACTGCT | -9720 |
| MmP5_354-811F | GGCTTTGTGACTGCCCCAT | MmP5_354-915R | AAGCCAGGGAGCTGTGACAG | -9050 |
| MmP5_353-526F | GGCAGCCATGGTAGAAGGAA | MmP5_353-628R | GGCCCCTGTCTGCTAGCTT | -7765 |
| MmP5_352-276F | CCGGCACTGTGAAAGTGCTT | MmP5_352-376R | CTCCCCCAACAAATGCAAAT | -6515 |
| MP5_49-148R | TGTCAGAGAAGGCTGTTTTTCCA | MP5_49-42F | ACCTAGCCCGACCTTTTGTCTT | -6 027 |
| MmP5_351-161F | CTCGGACTTTCAGTGGATACATGT | MmP5_351-262R | GGACCCCAGCTTTACTCTCTTACTC | -5400 |
| MmP5_350-207F | GCAGACTGGGATCCTTTGGA | MmP5_350-307R | ACGTCTGCCATGTGTTCCTG | -4446 |
| MmP5_349-410F | GTTCTGACCCCGCGATCTAC | MmP5_349-510R | CCAAGCGGCGACCAAGTAT | -3649 |
| MmP5_348-533F | CGCGCTTTCAACTTCTCCA | MmP5_348-634R | CCGTGTCATCCGAAGTGGTT | -2772 |
| MmP5_347-329F | GAGGCTGGGCTGCAACCTA | MmP5_347-429R | TGGCGAAATCTGCTCAGTGA | -1568 |
| MP5_46-148R | GCTCTCTCCCCTAGGTTGCA | MP5_46-39F | GGACAATGGCAGTTTCGGAA | -1 212 |
| MmP5_346-776F | TCTAGGTCCCATTCAGCCACC | MmP5_346-879R | CCGGGTTTTATCAGCAGCTTT | -885 |
| MP5_23-104F | CGAAGGCACCGTGAAATGAT | MP5_23-241R | GGTTCTTGGCCCGCAGT | 0 |
| MmP5_345-308F | ACACATGCTAATATGCTCAAGGCA | MmP5_345-408R | GCAGGAGTTTGTGTTACCCCA | 453 |
| MmP5_344-582F | ACTGTGCCAAGAAGGCCTGT | MmP5_344-683R | GGTGCTGCTCCAATGAAGGA | 1179 |
| MmP5_343-664F | AGGCCTACAAGTTCAGGGCA | MmP5_343-770R | TCCAAGTCCTGAGGTCCAGC | 2097 |
| MmP5_342-492F | CCTTCATCTGCAACCTGCGT | MmP5_342-592R | TTTCCACTCCCTGCACCTTC | 3269 |
| MmP5_341-187F | CACCGTGGCAGTTGTAGGAGA | MmP5_341-287R | AGAGGTCTTGGGTTTTGGTGG | 4574 |
| MmP5_340-646F | GGACCAGGTTGCCTCAAGTACA | MmP5_340-746R | GCTTTGAGCTAAGAGCATCACCA | 5613 |
| MmP5_339-281F | CGCCAGGTCCTTCTTGTTTC | MmP5_339-381R | TGTAACTTCGTCGGAGACGTCA | 6483 |
| MmP5_338-343F | GTCTTGCCTAGTCCCATGGC | MmP5_338-443R | TGGAGGGCAGGAAGTTTGAA | 7309 |
| MP5_42-121R | ACACTGTAAGCACGACCCGTT | MP5_42-1F | AGGACTTAGTATCTTACCCCGCACT | 7 400 |
| MmP5_337-415F | AATCTTGTCGCTGCTCGCTC | MmP5_337-516R | AGGTTTGCTTAGGCAGCCAA | 8346 |
| MmP5_336-385F | TCAGGTATGACACTGGCCACA | MmP5_336-485R | ACCAAGCTCACTGGCACAGA | 9376 |
| MmP5_335-621F | GAGGGCTCATCTGCCAGGA | MmP5_335-721R | TTTCCACCGTTTCTGTGCC | 10140 |
| MmP5_334-260F | TTGGCAACTCAGAGGCTAAGGA | MmP5_334-370R | GATATATGTGTGACCTTGGCGGT | 11601 |
| MmP5_333-157F | CTGACACCTTGATGGGCAAGTT | MmP5_333-259R | GACATGGAGGAGTGAATCAGCTT | 12804 |
| MP5_16X02-170R | GACGGCCACTCCCAGATGTA | MP5_16X02-28F | CTGGAGCCCGTGCCTTTAC | 13 175 |
| MP5_29-193R | GACAGAGTGGCATTCGGAAGTTA | MP5_29-72F | GACATTTTGTCCACTGTGGTTCTT | 15 281 |
| MP5_44-176R | TGGTCTTTCATGCCTCTCTTCTCT | MP5_44-76F | AGGACATTTCCATGCTATCAGCTT | 17 528 |
| MP5_41X03-154R | CAAAGGTTGCCACTCCCAAA | MP5_41X03-45F | ACAGACTCGCTCTGCCAACA | 18 847 |
| MP5_05-105R | TCTATAACCAGCCTTCTGCGATG | MP5_05-5F | AGCGAAGTGAGAGCAAGGGA | 28 316 |
| MP5_51X09-109R | CACCTACTGCAGGGAGTGAATTT | MP5_51X09-4F | TACTCACCAAGCAGCCCTGG | 31 313 |
| MP5_27-155R | TGGACAGAGCTGCCAGGAC | MP5_27-26F | CCCTTCCAGGCTCAGAGCTA | 46 432 |
| MP5_03-602F | CCATTTCACTGGCTTATTGCTTC | MP5_03-704R | CAGGCAGTGGCTGATCTCTGT | 53 943 |
| MP5_10-181F | AGTGCTGGGCTTAGTCAACCAT | MP5_10-299R | CTGAAATTTGAAGCCGGCAT | 59 772 |
| MmPAX5_286-460F | TATGGGTATGAGCCACATGAAGA | MmPAX5_286-561R | CCTGGAGGGTCAGTCCTGC | 59802 |
| MmPAX5_285-724F | GGGAAGCTGTGTTTGGACCA | MmPAX5_285-829R | AGGTTTCTGTGGCCTCGGA | 60538 |
| MmPAX5_284-609F | AGGGTGGCTTGACTCTAGATTCC | MmPAX5_284-710R | CATAACTTTGGTTGGCTCCATG | 61653 |
| MmPAX5_283-559F | CCCAGGCCTTATTGCAAGAA | MmPAX5_283-659R | AGAGCACGGCTCCTTGGTT | 62703 |
| MmPAX5_282-316F | CCTCGTTTAATGTCCTCACCCT | MmPAX5_282-423R | TTTGTCAAGTCACCTAGCACAGGT | 63946 |
| MmPAX5_281-554F | CCGGAAGTGAGTGGCCATT | MmPAX5_281-654R | TCTCTCCTTTGGTGGTGGGA | 64708 |
| MP5_32X06-195R | GCTCACCAAAGGTATTCAGGAGTC | MP5_32X06-66F | AAACACGCGGTCCAGCAC | 65 137 |
| MmPAX5_280-177F | TGGGATGCCTCACAGTGAAC | MmPAX5_280-280R | TGGCCATAGGCTCTGAGCA | 66085 |
| MmPAX5_279-388F | CTTGGCTTGCTGGCAGATG | MmPAX5_279-488R | CTTGTGACTCCTCAGAGCGGT | 66508 |
| MmPAX5_278-599F | GTCTCCATGGCCCAAGTCA | MmPAX5_278-700R | ATCACCAGATTAGGGTCAAGCTG | 67663 |
| MP5_06-228F | GAGGCTGCTCCAGCTGATTTAT | MP5_06-362R | AACACCAGGCTATTAATCTGGCTT | 68 640 |
| MmPAX5_277-316F | TGACTGCCTGTCACGCAGAT | MmPAX5_277-423R | TGGTACCGTTGTCTGGAAAGAGT | 68946 |
| MmPAX5_276-660F | ACACACTGGCCACGACATGA | MmPAX5_276-760R | TGTGAGTCAGCTATGGCTCCA | 69602 |
| MP5_09-163R | TTGTGTTGGACGATTAAACCTCAT | MP5_09-30F | AGCCCACTTGAGGCAATTGT | 87 486 |
| MP5_24-157F | CCAGCTTTCCAGAAGCCAGT | MP5_24-262R | GCATGGGAGGCAGCTTTGT | 88 850 |
| MP5_47-112R | CTGTTTCTTCTCTCCTCCATGCTAT | MP5_47-7F | TGAGTCCCTATGAAATTACCTGGAA | 95 184 |
| MP5_52X07-149R | CTTCTCCAACCCAGCACCAT | MP5_52X07-27F | CCAGGAGTCCCCACTGTCTGT | 100 997 |
| MP5_18-161F | ACATCCCGGTCATCAATTTCA | MP5_18-264R | TGTTCAATCTCCCCTCGTGTT | 119 045 |
| MP5_50-132R | GCAAACCACATGAGATCCCAT | MP5_50-14F | TGCAATCACACAGCTTCCTGA | 121 011 |
| MP5_40-144F | CATTTTGCTACCAGCTCGGTT | MP5_40-244R | TGAAGCAAGGGTTGGAGGATT | 132 260 |
| MP5_43-165R | TGTGTTTTGTTGCTTTGATTCTCC | MP5_43-34F | TCACCAGGCACCATCCCT | 140 730 |
| MP5_12-271R | CCTCATGTCTTCCCTTTCAGTTG | MP5_12-166F | TTTCCAGCCTCCCCAGC | 149 662 |
| MP5_11-315F | CTGCGAAGGAACAATGCTTGA | MP5_11-420R | CCTGAATGGCTGCCTGCTT | 152 332 |
| MP5_38-148R | GTCTAGTCTCCTCGCAAACACTTG | MP5_38-37F | TGCTGGGAAGTCACAGTGCT | 159 315 |
| MP5_34-158R | GGTGGGAGCGGACCATCT | MP5_34-46F | CAGGCAAACAGGCTCCACA | 164 932 |
| MP5_53X10-166R | CACACTCCCCTGAAACGTCCT | MP5_53X10-56F | CTGGAGAAGCTCATCAAGCGA | 173 311 |
| MP5_54X05-193F | ATGCGCAGCCATCTCTTACCT | MP5_54X05-293R | GCGGGCTCCTCATACTCCA | 179 255 |
| MP5_37-101F | TTGGTTGGAATGTTCAACTCTCC | MP5_37-239R | TTATAGACGGTGCGTTTCCCTT | 184 326 |
| MP5_20-223F | TCACAAGGGACAGTCACTCATGA | MP5_20-358R | TGTTGAATCTGGGATTTGTGGAT | 185 917 |
| MP5_36-130R | AGAGATGGAGTGGGCAAGTGA | MP5_36-19F | TTGTCGTCATCATCCATTGCA | 187 810 |
| MP5_22-139F | AGTCCGGAGGCTCTGCACT | MP5_22-239R | TTCTGCTTGGCTGAGTTTGCT | 191 745 |
| MP5_39-520F | ATCTGTTGGCTGGGATAAGACCT | MP5_39-622R | TGGGTTTGTATGCCGGCT | 199 068 |
| MP5_13-179R | TGTCAAATAGCTTCTCAGATGCATT | MP5_13-71F | GTGAATCAAAGTGGCTTCTTGCT | 199 942 |
| MP5_08-276F | CACTTCAGCTTCCCATGTAATCAT | MP5_08-378R | CCTAACGAGCTGCGTGTCACT | 202 036 |
| MP5_30-134R | TCTGCTAAAATGCTAATGATCGCT | MP5_30-34F | TCAGCTGACGAGTTATAAGCCATG | 213 367 |
| MP5 _21-361F | GAGATGCCTGACTTATGCGGA | MP5 _21-463R | AGGAAATCACACCCGACTCAA | 223 984 |
| MP5_33-120R | CCAGCTGTGCTGGCTCATT | MP5_33-14F | CAGCCAAACCAATCACCCTT | 253 273 |
| MP5_31-109F | CCTCTCTGGGCTTAAATGAGTCTC | MP5_31-219R | GGCAAAGATATGCGCAATCAA | 256 533 |
| MP5_02-245F | AGCAGGGATTGATGGCAGTC | MP5_02-370R | AATGTCCTGGAATGCAGATGC | 259 682 |
| MP5_28-124F | GGAAATCTGGATGGGAAGCA | MP5_28-224R | GCCTGTTCTTCTGCCCTTGA | 265 301 |
| MP5_45-21F | AGCGTCTTCTCCCATGTCGA | MP5_45-127R | AGGAATCCCTCGTAGTAACATTTCA | 275 885 |
| MP5_26-184R | ACCAGAGGAGTCATAGCTGAGTGA | MP5_26-82F | AGAGCAGGTGGATTGCTTCAA | 281 636 |
| MP5_35-166F | GAGGAGCTATCATCAGGCGAGT | MP5_35-274R | GAGACCCGGCTGACAGGAT | 285 352 |
| MP5_48-167R | ATTCCGCCAGCACGCTT | MP5_48-64F | TGCAACTTGGCCTTGACGA | 286 096 |
| MP5_19-101F | GCTGCTGCTTTTCCCCAGTAT | MP5_19-212R | GCGCTTGGTTAGACAAATGGA | 306 906 |
| MP5_01-134F | GGTCTGCAAATGGCCACATA | MP5_01-245R | GCGCTGTCAGGCTCACAAT | 318 047 |
| MP5_14-254F | CGATACGGTGCAGGGAGAA | MP5_14-359R | AATGTGTGAGCTGCGCTTCA | 330 582 |
| MP5_04-281F | CGCCTAGCCCTGCCAGAT | MP5_04-420R | TTATCCAGAGCCCAGCACATC | 333 458 |
| MmABL1_Pr-36F | TGGTCACAGTGCAGCCCA | MmABL1_Pr-139R | GAAGAAATGCATGACCAGCAACT |  |
| MmGUSB_Pr-190F | GACCCAACACGCACTCCATT | MmGUSB_Pr-297R | TCTTCCAGGTCCCGCCTC |  |

Position compare to the beginning of exon 1A is indicated.
